# 병원 내 언어폭력에 노출된 임상 간호사의 경험: 현상학적 연구

**DOI:** 10.4069/kjwhn.2022.05.24.2

**Published:** 2022-06-29

**Authors:** Min Soo Woo, Hyoung Suk Kim, Jeung-Im Kim

**Affiliations:** 1 Graduate School, School of Nursing, Soonchunhyang University, Cheonan, Korea; 1 순천향대학교 간호학과 박사과정; 2 School of Nursing, Soonchunhyang University College of Medicine, Cheonan, Korea; 2 순천향대학교 의과대학 간호학과

**Keywords:** Nurses, Qualitative research, Verbal behavior, Workplace violence, 간호사, 질적 연구, 언어적 행위, 직장 내 폭력

## Abstract

**Purpose:**

This study aimed to describe clinical nurses’ lived experiences of workplace verbal violence through qualitative research using descriptive phenomenology.

**Methods:**

Six female Korean nurses who had less than 5 years of clinical experience and had experienced verbal violence in the workplace within the past year participated in the study. Data were collected through one-on-one in-depth interviews with the participants and analyzed using Colaizzi’s phenomenological method.

**Results:**

A total of 27 codes, eight themes, and four theme clusters were derived from the participants’ statements. The four theme clusters of the clinical nurses’ experiences of verbal violence in the workplace were as follows: “tip of the iceberg,” “beyond me and my control,” “fear and resignation,” and “personal burden.” The participants recognized that nurses experienced verbal violence daily, and that the causes of and responses to verbal violence were determined by external situational factors rather than nurses’ individual problems. This suggests that nurses felt that they had no choice but to personally cope with verbal violence and bear the consequences due to systematic indifference and silence about verbal violence experienced by clinical nurses.

**Conclusion:**

The findings show that verbal violence was pervasive and unmerited, yet often endured at the cost of a personal burden to nurses. A clear definition of verbal violence and education for employees are needed, and a reporting system should be established to report all forms of violence regardless of the severity of the incident.

## Introduction

언어폭력은 임상 간호사들이 가장 많이 경험하는 직장폭력의 한 형태로[1], 반복될 경우 피해자에게 회복하기 힘든 영향을 미칠 수 있다[2]. 직장 내 언어폭력은 피해 간호사들에게 외상 후 스트레스장애, 결근, 이직 의도를 높여 사직을 초래할 수 있으며[3], 사회적, 상황적, 개인적 특성과 결합하여 물리적 폭력으로 확대될 수 있다[4,5]. 또한 선행 연구[6]에 따르면, 환자에게 언어적 공격(verbal aggression)을 당한 정신과 간호사들이 환자에게 억제대나 구속복을 더 자주 적용하는 경향이 있는 것으로 나타나는데, 이는 간호사들이 경험하는 언어폭력이 환자에게 제공되는 간호의 질에도 영향을 미칠 수 있음을 보여준다.

이에 미국 직업안전위생국(Occupational Safety and Health Administration, OSHA) [5]에서는 심각한 신체적 손상을 초래할 수 있는 물리적 공격이나 위협뿐만 아니라 직장 내 언어폭력에도 관심을 가질 것을 촉구하고 있다. 그러나 임상 간호사들에 대한 언어폭력 등 직장 내 폭력 사건은 잘 보고되지 않는 경향이 있고[1,2], 직장 내 언어폭력에 대한 정의나 이를 지칭하는 용어도 다양하다[4]. OSHA [5]는 직장 내 언어폭력(workplace verbal violence)을 언어적 학대(verbal abuse), 위협(threats), 적대감(hostility), 괴롭힘(harassment)를 포함하는 개념으로 정의하고 있으며, 세계보건기구[2]는 언어적 학대, 위협, 따돌림(bullying/mobbing), 괴롭힘을 심리적 폭력(psychological violence)으로 분류하고 있다. 국내 연구에서 직장 내 언어폭력은 “환자, 보호자, 의료인 등 다른 사람들로부터 부당하게 받은 욕설, 반말, 고성, 협박의 개념”[7], “부당하고 폭력적인 말을 사용해서 상대방에게 정서적인 강제력을 행사하여 정당한 이유 없이 굴욕감을 주고 품위를 떨어뜨리는 행위”[8] 등으로 정의되는데, 같은 정의를 사용하는 경우에도 영어로는 언어폭력(verbal violence) [7,8], 언어적 학대(verbal abuse) [9,10]가 혼용되고 있다. 국내 임상현장에 적용되고 있는 고용노동부의 「고객응대근로자 건강보호 가이드라인」[11]과 「직장내 괴롭힘 판단 및 예방, 대응 매뉴얼」[12]에서도 직장 내 폭력, 특히 언어폭력이 명확하게 정의되어 있지 않은 실정이다.

이러한 개념의 모호함은 문제의 본질을 파악하는 데 장애가 되고 해결 방향에 혼란을 가져올 수 있다. 그러나 임상 간호사들이 경험하는 언어폭력에 대한 최근의 국내 연구는 언어폭력 경험이 간호사들에게 미치는 영향을 파악하는 양적 연구가 주를 이루고 있다. 임상 간호사들이 경험하는 언어폭력에 대한 질적 연구들 또한 중환자실 간호사[13], 응급실 간호사[14], 정신보건 간호사[15] 등 특정 대상자에 국한되거나 직장 내 폭력 경험의 일부[16]로만 다루고 있어 관련 현상을 심층적으로 이해하기에는 한계가 있다. 한편 연령이나 임상경력이 낮은 간호사가 직장 내 언어폭력에 더 많이 노출되며[17,18], 여성 간호사가 남성 간호사보다 언어폭력을 더 많이 경험하는 것으로 나타났으나[19], 이러한 특징을 반영하여 임상 간호사들이 경험하는 언어폭력 현상을 심층적으로 탐색한 질적 연구는 부족한 상황이다.

Colaizzi [20]의 현상학적 연구는 심리적 경험을 직접 관찰할 수는 없지만 지각적으로 기술할 수 있다는 사실에 근거하고 있으며, 간호사들의 생생한 경험을 통해 병원내 언어폭력 현상을 깊이 있게 이해할 수 있게 해 준다. 또한 연구자가 분석한 맥락적 의미가 왜곡되지 않았는지에 대한 참여자의 검증과정을 거치므로 연구자의 선입견을 여과시킬 수 있다는 장점이 있다.

이에 본 연구는 Colizzi [20]의 현상학적 연구방법을 적용하여 임상 간호사들의 언어폭력 경험에 대해 심층 면담하고 그들의 관점에서 언어폭력 경험의 의미와 구조를 탐색하고자 한다. 병원에서 일하는 임상 간호사들이 업무 중에 경험한 언어폭력의 구조와 의미를 총체적이고 심층적으로 기술하여 임상 간호사들이 경험하는 언어폭력의 사회적, 조직적 맥락을 파악하여 실효성 있는 해결 방안을 모색하여 궁극적으로 간호사가 근무하기에 적합한 근무환경을 만들어가는 기초 자료로 활용되기를 기대한다.

## Methods

Ethics statement: This study was approved by the Institutional Review Board of Soonchunhyang University College of Medicine (1040875-201909-SB-053). Informed consent was obtained from the participants.

### 연구 설계

본 연구는 질적 연구설계로서, 임상 간호사가 겪은 언어폭력에 대한 체험의 구조와 의미를 탐구하고자 하였다. Colaizzi [20]의 현상학적 연구방법은 개인의 속성보다는 연구 참여자 전체에게 나타나는 공통적인 속성을 이끌어내는 데 초점을 두므로 본 연구의 언어폭력의 경험에 대한 구조와 의미를 파악한다는 목적에 적합한 방법이다[21]. 본 연구는 SRQR (Standards for reporting qualitative research: a synthesis of recommendations) 가이드라인(https://www.equator-network.org/reporting-guidelines/srqr/)에 따라 기술하였다.

### 연구 대상

#### 연구 참여자 선정

임상 간호사의 언어폭력 경험을 최대한 풍부하면서도 구체적으로 기술하고자, 면담 당시 임상 경력 5년 이내의 여성 간호사이면서 최근 1년 이내에 언어폭력을 경험한 사람 중에서 의도적으로 참여자를 표집하였다. 병원에 근무하는 연구자들의 지인들에게 연구의 취지와 목적을 설명하여 자신의 언어폭력 경험을 생생하게 이야기해 줄 의향이 있는 간호사를 소개받았고, 그 중 병원 규모나 부서 등 간호사의 특성과 가해자를 모두 고려하여 참여자를 선정하였다. 총 6명의 간호사를 면담하였으며, 연구 참여자의 일반적 특성은 Table 1과 같다.

#### 연구자 준비와 성찰

연구자들은 대학원 박사과정에서 공동연구자의 질적 연구 방법론을 수강하였고, 질적 연구 세미나에 참석하여 질적 연구 수행을 위한 역량을 쌓았다. 교신저자는 질적 연구방법을 적용하여 박사논문을 작성한 바 있으며, 공동 연구자는 질적간호연구학회의 평생회원으로 여러 편의 질적 연구 논문을 출판한 바 있다. 연구자들은 자료 수집부터 분석, 기술에 이르는 전 과정에서 간호사의 언어폭력 경험에 대한 연구자의 선이해와 편견을 배제하기 위해 의식적으로 노력하면서 성찰적 태도를 견지하였다.

### 자료 수집

자료 수집은 2019년 10월 12일부터 12월 21일 사이에 일대일 심층면담을 통해 이루어졌다. 면담은 조용하고 부드러운 분위기의 카페, 연구실, 강의실 등 다른 사람의 방해가 없고 참여자가 선호하는 장소에서 이루어졌다(평균 소요 1시간 20분). 모든 면담은 “지난 1년 동안 병원에 근무하면서 겪은 언어폭력 경험에 대해 이야기해 주시겠습니까?”하는 개방형 질문으로 시작하였고, 면담 중에 나타난 중요한 내용과 관련하여 의미를 명확히 하기 위해 면담 상황에 따라 언어폭력이 일어난 상황, 언어폭력 경험 후의 신체적∙정신적 증상, 개인 및 조직의 대처 등에 대해 보다 구체적으로 설명해 줄 것을 요청하는 질문을 추가하였다. 연구자는 참여자가 자신의 경험에 대해 충분히 생각하고 이야기하도록 격려하며 경청하였고, 종결 단계에서는 면담 내용을 요약 정리하여 참여자의 확인을 받고 더 나누고 싶은 이야기가 있는지 확인하였다.

연구자는 면담 중에 현장 노트를 작성하여 참여자의 감정을 확인할 수 있는 비언어적 표현들까지 기록하였고, 모든 면담은 참여자의 동의 하에 녹음하였다. 면담을 마친 직후 각 연구자들이 직접 필사하면서 녹취록을 작성하였다. 녹취록은 코드화된 일련번호를 부여하여 개인 식별 정보가 구별되지 않도록 작성하였으며, 잠금 장치가 있는 곳에 보관하여 연구자 외에는 열람하지 않도록 관리하였다. 필사본을 작성하는 과정에서 의미가 명확하지 않은 경우 추가 면담을 진행하였다. 참여자 6명 중 4명은 1회씩, 1명은 2회 면담하였고, 또 다른 참여자 1명은 대면 면담과 전화 면담을 각각 1회씩 추가하였다. 먼저 면담한 자료를 연구자들이 함께 모여 검토하면서 면담과 분석을 병행하였고, 더 이상 새로운 자료가 나오지 않았을 때 자료가 포화된 것으로 판단하여 면담을 종료하였다. 면담 중 참여자가 힘들어하는 경우 감정을 충분히 표현할 수 시간을 주고 정서적으로 지지하였으며, 참여자가 원할 경우 전문가 상담을 받을 수 있도록 지원할 수 있음을 알렸다.

### 자료 분석

자료 분석은 현상학적 접근방법 중 참여자로부터 기술된 내용에서 명확한 의미를 찾고 현상의 본질을 정확하게 진술하도록 하는 Colaizzi [20]의 절차를 따랐다. 연구자들이 함께 원자료를 보면서 코딩하고 결과를 도출하였다. 자료 분석 절차는 다음과 같다.

첫 번째 단계로 녹음내용을 반복해서 듣고 필사본을 여러 번 읽으면서 참여자들의 경험에 대한 전체적인 느낌을 이해하고자 하였다. 두 번째 단계로 참여자들 사이에 반복되는 진술, 강조되는 내용, 연구 참여자들의 직장 내 언어폭력 경험의 의미를 잘 드러낸다고 판단되는 내용에 밑줄을 치면서 본 연구의 관심 현상에 대한 의미 있는 진술과 구절을 확인하였다. 세 번째 단계는 의미형성 단계로 중요한 진술들의 의미를 확인하여 연구자의 언어로 바꾸고 코드화(codes)하여 엑셀 파일에 정리하였다. 네 번째 단계로 앞 단계에서 확인된 의미들을 모든 참여자의 진술에서 공통되는 주제들(themes)로 묶으면서 분류하였다. 다섯 번째 단계로 4단계에서 생성된 모든 주제들을 주제모음들(theme clusters)로 통합하여 임상 간호사들이 경험하는 언어폭력 현상에 대한 포괄적인 설명을 작성하였다. 여섯째 단계로 임상 간호사의 언어폭력 경험에 대한 포괄적인 기술을 독자들이 잘 이해할 수 있도록 진술을 명료하게 정리하였다. 마지막으로 참여자 2인에게 분석 결과를 보내어 분석 결과가 참여자의 경험을 잘 반영하고 있는지 확인받는 절차를 거쳤다.

### 연구의 엄격성과 확실성 확보

Guba와 Lincoln [22]이 제시한 기준에 따라 연구의 엄격성과 확실성을 확보하고자 하였다. 연구의 사실적 가치(truth value)를 충족하고자 가능한 가까운 시일 내 환자나 보호자, 의사, 동료 간호사에게 직접 언어폭력을 당하고 이에 대처한 경험이 있어 현상을 생생하게 진술할 수 있는 참여자를 선정하였다. 자료의 왜곡이나 누락이 발생하지 않도록 최대한 면담이 끝난 직후에 바로 필사를 진행하였고, 필사 자료와 녹음 내용을 반복적으로 검토하면서 참여자의 진술을 그대로 반영하도록 노력하였다. 또한 최종 분석 결과가 연구 참여자의 경험을 잘 반영하고 있는지 확인하는 절차를 거쳤다. 적용성(applicability) 확보를 위해 풍부한 자료를 수집할 수 있도록 근무 병원, 근무 기간, 근무 병동이 다른 참여자를 의도적으로 선정하였다. 또한 면담 과정에서 참여자가 최대한 편하게 자신의 경험을 진술할 수 있도록 조용하고 독립된 공간을 확보하고, 연구자의 개입을 최소화하면서 경청하여 참여자 스스로 경험을 이야기할 수 있도록 유도하였다. 연구의 일관성(consistency)을 유지하기 위해 연구자들은 Colaizzi 분석법에 충실히 따르고, 주기적으로 18회 이상의 연구회의를 통해 연구의 주질문에 집중하면서 자료 수집과 분석 과정을 병행하며 순환적으로 진행하였다. 또한 연구의 전체 진행 과정을 기록하고, 연구 제에 대한 연구자의 선행 지식이나 편견이 연구 참여자의 경험을 왜곡하지 않도록 의식적으로 노력하면서 중립성(neutrality)을 유지하고자 하였다.

## Results

연구 참여자들의 의미 있는 진술 349개를 92개의 구성된 의미로 분류한 후 27개 코드, 8개 주제, 4개 주제모음을 도출하였다(Table 2). 최종 도출된 주제모음은 ‘빙산일각’, ‘상황귀인’, ‘두려움과 체념’, ‘개인부담’이며, 구체적 내용은 다음과 같다.

### 주제모음 1. 빙산일각(氷山一角)

이 주제모음은 ‘언어폭력으로 인식되는 상황이 다양함’, ‘너무 흔해서 무감각해짐’이 포함되었다. 참여자들은 병원에서 일어나는 언어폭력의 양상이 다양하고, 가해자도 환자 및 보호자, 동료 간호사, 의사 등 다양하다고 하였다. 또한, 참여자들은 간호사들이 일상으로 여길 정도로 언어폭력이 빈번하게 발생하여 이에 대해 무감각해질 정도라고 하였다. 그럼에도 불구하고 공론화되거나 보고되지 않는 경우가 많아, 드러난 언어폭력은 빙산의 일각에 불과한 것으로 나타났다.

#### 주제 1: 언어폭력으로 인식되는 상황이 다양함

이 주제에는 ‘무시당하고 모욕감을 느낌’, ‘억울하게 화풀이 당함’, ‘부당하게 질책 받음’, ‘의도된 괴롭힘을 당함’, ‘위협을 느낌’이 포함되었다.

참여자들은 가해자들의 의도는 모르지만, 간호사 호칭을 제대로 부르지 않는 것, 자신을 무시하는 듯한 태도, 작성자를 밝히지 않은 선배의 질책성 메모, 환자나 보호자들이 간호사를 대할 때와 의사를 대할 때의 태도 차이를 ‘무시나 위협’의 신호로 느끼고 언어폭력으로 인식하였다. 또한 소리 지르면서 멱살을 잡으려고 하거나, 인격 모독적으로 말하면서 볼펜, 서류를 집어 던지고 의자를 발로 차는 등 위협적인 행동이나 물리적 폭력을 동반하는 경우도 흔하다고 하였다. 참여자들이 느끼는 언어폭력은 언어적, 비언어적 표현을 포함하고, 잘못된 호칭부터 신체적 위협에 이르기까지 광범위한 양상을 보이고 있었다.

*(제가 신규도 아닌데) 제 얼굴 처음 봤다는 이유로 (환자가) ‘야! 핫바리 데리고 오지 말라고. 나는 임상 실험 대상이 아니다. 다른 사람 데리고 와라.’라고‧‧‧ (환자에게 저희가) 아랫사람들이라는 인식이 (있는 것 같고)‧‧‧ 호칭도 그냥 ‘아가씨, 언니’ 이렇게 부르는 사람이 엄청 많아요. (참여자 1)*

*다른 (의사)선생님들은 환자한테 화나는 상황들, 환자가 하는 행동에 화나거나 그러면 (간호사들과 통화하면서) 전화로 씨X, X같네. 이렇게 말하는 사람들도 많고. 반말은 밥 먹듯이 하고‧‧‧ (참여자 5)*

*(전공의가 교수님한테) 혼이 났다고‧‧‧ (저한테) 따지러 왔다는 거예요‧‧‧ 저는 의사가 하라는 대로 했고‧‧‧ (전공의 처방이) 너무 이상해서 세 번이나 물어보았는데‧‧‧ (저보다) 윗 연차 선생님이나 수간호사 선생님은 잘못한 의사에게는 아무 말도 안 하시고, 저한테 엄청 뭐라고‧‧‧ (참여자 4)*

*‧‧‧(선배 간호사들이) 바빠서 태우는 것보다는‧‧‧ (괴롭힘을 당하는 간호사가 선배들의) 타겟이 돼서 더 그러는 거 같아요‧‧‧ 얘(타겟이 된 간호사)가 싫어서 딴 사람한테는 그냥 넘어갈 수 있는 것들을 얘한테는 걸고 넘어지는‧‧‧ (괜히) 첫 인상이나 하는 행동들이 좀 마음에 안 든다거나 하는 (이유로 그러는 것 같아요)‧‧‧ (참여자 5)*

*(선배 간호사에게) 잘못한 것을 혼날 때도 인격 모독적으로 뭐라고 하는 사람들 있잖아요. ‘너는 이런 머리로 대학병원을 들어왔어?’라고‧‧‧ 말을 함부로 하는 식으로 자기 기분 나쁜 것을 직접 표현하면서 볼펜을 집어 던지기도 하고‧‧‧ (참여자 4)*

#### 주제 2: 너무 흔해서 무감각해짐

이 주제에는 ‘일상적으로 폭언에 노출됨’, ‘웬만해서는 이슈가 안됨’, ‘다양한 가해자’, ‘폭력을 폭력으로 인식하지 못함’이 포함되었다.

연구 참여자들은 언어폭력이 너무 흔해서 만성화되었고, 동료들도 이를 사소한 일로 치부하는 분위기가 팽배했다고 하였다. 언어폭력의 가해자로는 환자, 보호자, 동료 간호사, 의사, 불특정 가해자까지 다양하였다. 이 중 유난히 폭력적인 동료나 누구도 통제할 수 없는 사람도 있지만, 가해자들이 자신의 행위가 언어폭력인 줄도 모르고 행하는 경우도 있다고 하였다. 특히 참여자들은 태움에 비해 환자나 보호자에 의한 폭력 피해는 사회적 이슈가 되지 않아서 전반적으로 간호사가 경험하는 언어폭력의 심각성에 대해 인식이 부족하다고 느끼고 있었다.

*‧‧‧(언어폭력을 당해도) 그러려니 하고 상처받아도 넘기고 하는 그런 거에 만성화된 거 같다는 거‧‧‧ 너무 많아서‧‧‧ 말씀드린 조금 컸던 사건들 말고도 너무 비일비재해서 이제 사소한 거는 그냥 (넘겨요)‧‧‧ (참여자 6)*

*(내가 선배 간호사에게 뭘) 물어보면 대꾸 안 하고, (선배 간호사가) ‘야, 야 이것 좀 해’라고 말하면 (나는) 하던 일 다 미뤄두고 해야 되고, (선배 간호사가) 전화해서 ‘이거 했어?’라고 해서 (내가) 대답하면 (선배는) 대답 안 듣고 전화 끊고‧‧‧ 근데 그 선생님(선배 간호사)은 ‘내가 너한테는 잘해 줬잖아‧‧‧’ 그렇게 말씀을 하세요. 병원 그만 둔 지금도 (그 선생님에게서) 가끔 연락 와요‧‧‧ 가해자(인 그 선배 간호사)는 그걸(자신이 내게 언어폭력을 가했다는 사실을) 잘 모르고. 제가 그 선생님을 싫어하는 걸 모르는 거 같은데‧‧‧ (참여자 3)*

*‧‧‧(환자, 보호자 등에게) 그런 일이 있었다고 해서 언론에 공개되거나 그러지는 않잖아요. (간호사 조직 내에서) 일반적인 태움이 있다고만 나오지‧‧‧ (참여자 1)*

### 주제모음 2. 상황귀인(狀況歸因)

이 주제모음에서는 ‘원인이 내 탓이 아님’, ‘내 통제를 벗어남’의 2가지 주제가 포함되었다. 참여자들은 언어폭력이 발생하는 원인이 개별 간호사의 행위에 있지 않고 구조적이고 상황적인 요인에서 비롯되기 때문에 해결책 또한 조직적이고 사회적인 차원에서 접근해야 한다고 인식하고 있었다.

#### 주제 3: 원인이 내 탓이 아님

참여자들은 언어폭력이 환자나 보호자의 간호사에 대한 잘못된 인식, 환자 요구를 우선시하기 때문에 무조건 들어줘야 하고 여러 사람들 사이에서 의사소통하며 일하는 직무 특성, 그리고 상황을 악화시키는 근무환경 때문에 발생하는 것으로 인식하고 있었다. 참여자들은 환자, 보호자의 특성에 따라 간호사 자신의 잘못과 상관없이 화풀이 대상이 되는 등 언어폭력에 더 취약할 수밖에 없다고 하였다. 또한, 간호사의 인력이 부족한 상황에서 충분한 간호를 제공할 수 없을 때 더 언어폭력이 발생하는 것으로 설명하였다.

*환자들도 간호사를 의사보다 밑에 있는 사람이라고 생각하는 것 같아요‧‧‧ 의사에게는 어쩔 줄 모르시면서 저희에게는 반말, 아가씨, 어~~이 이렇게 부르시는데, 그런 거 보면 환자들은 의사들보다 간호사들을 만만하게 보는구나‧‧‧ ‘간호사가 뭘 알아. 의사가 시키는 대로 하면 되지.’하는 말도 (들었어요)‧‧‧ (참여자 4)*

*3교대니까‧‧‧ 내가 온전히 해결하고 다음 날 내 일이 되는 게 아니라 일이 (다른 사람에게) 넘어가고 그런 관계여서‧‧‧ (일을 넘겨받는 간호사는) 많이 예민해지고‧‧‧ 그 일들이 주치의가 빨리빨리 (해결)해주면, 한 번에 (해결)하면 좋은데‧‧‧ 전화를 4, 5번 해도 안 해주고‧‧‧ 그런 경우가 많고 그러면‧‧‧ 서로 바쁘니까 협조가 잘 안되고 (그래서 언어폭력이 발생하기 쉬운)‧‧‧ (참여자 5)*

*‧‧‧내가 그 환자를 잘 안 봐주고 싶어서 안 하는 것보다 진짜 시간이 없고 너무 바빠서‧‧‧ 진짜 아홉 시간 열 시간 일하는데, 1분도 안 쉬고 일할 때 정말 많거든요? 하루 종일 뛰어다니고 그렇게 일했는데‧‧‧ ‘너 진짜 이거 밖에 안 돼?’라고 들으면‧‧‧ 아‧‧‧ 이렇게까지 해야 되나, 하는 생각이 들어요‧‧‧ (참여자 6)*

#### 주제 4: 내 통제를 벗어남

이 주제에는 ‘내가 어찌할 수 없는 문제’, ‘혼자 감당할 수 없음’이 포함되었다. 참여자들은 느닷없이 언어폭력을 당한 경험이 많았으며, 원인이 명확한 경우에도 간호사 개인이 해결할 수 없는 문제로 화가 난 환자와 보호자, 동료 직원들에게 언어폭력을 당한다고 하였다. 참여자들은 언어폭력이 일어나는 상황에 압도되어 어찌할 바를 모르고 위축되어 상황을 통제할 자신이 없음을 표현하였다. 특히 동료 간 언어폭력을 경험한 간호사들은 대개 연차가 낮은 경우가 많았는데, 피해자인 간호사들은 스스로를 무력한 개인으로 보고, 가해자는 힘 있는 상급자나 조직 전체를 대표하는 것으로 인식하는 경향이 있었다.

*그 환자를 아예 처음 보는(담당하는) 상황이었고‧‧‧ (인계를 받지 못한 상태에서) 데이번이 없어서 제가 (담당의 전화를) 받은 거죠‧‧‧ (처방된) 대변검사가 안 나가 있어서 (화를 내는데)‧‧‧ 이건 어쩔 수 없는 거잖아요‧‧‧ 환자가 대변을 봐야 (검사를 나갈 수 있게) 되는 거니까‧‧‧ 전공의가 ‘이따위로 일 할래?, 똑바로 해라. 너네가 하는 일이 뭐냐.’‧‧‧ 전화를 계속해도 해결되는 게 없잖아요. (제가) 죄송하다고 하고 끊으려고 하는데도‧‧‧ 소리를 고래고래 지르는 거에요‧‧‧ (참여자 5)*

*신규 때는 제가 아무 말도 못했어요. 그냥 아예 대놓고 심한 말도 많이 들었어요. 왜 그렇게 말씀을 하시는지 저도 이해를 할 수 없었거든요. 근데 막 제가 한 조그마한 실수 가지고 자기네들끼리 저 있는 데서 말하면서 ‘환자 죽일 애다’ 이런 말도 많이 하고‧‧‧ 근데 그 사람들이 문제인 것 같아요. 저도 왜 그런지 알 수 없거든요‧‧‧ 저는 혼자고 그들(선배 간호사들)은 무리인데 그렇게 말하긴 힘들잖아요. (참여자 1)*

*저 혼자서는 (언어폭력 발생 시 대처가) 안될 것 같아요. 위 연차 분들이 솔선수범해야 하지 않을까 하는 생각이 들어요‧‧‧ 저보다 병원의 환경에 대해 잘 알고 계시는데, 그런 분들이 더 나서 주신다면 좋을 것 같다는 생각이 들어요. (참여자 4)*

### 주제모음 3. 두려움과 체념

이 주제모음에는 ‘두려워서 침묵함’, ‘체념하고 침묵함’이 포함되었다. 참여자들은 전반적으로 간호사들 사이에서는 언어폭력 문제에 대해 문제 제기 없이 웬만하면 참고 넘어가거나 침묵으로 대응하고 있다고 하였다. 그 원인으로는 주변으로부터 도움이나 지지를 받기보다는 비난을 받을 것에 대한 두려움, 경험상 잘 해결되기 어려울 것이라는 등의 이유로 인한 체념을 지목하였다.

#### 주제 5: 두려워서 침묵함

참여자들은 언어폭력을 경험한 간호사들이 자신들이 언어폭력을 당했다고 말할 경우 다른 간호사들이 지지해주지 않을 것이고, 소문의 주인공이 되는 등의 ‘후폭풍’을 감당할 자신이 없다고 하였다. 또한 문제 제기를 한 후 주변 사람들에게 민폐가 될까 두려워 침묵하고 있었다고 하였다.

*(언어폭력 피해를 당해도) 그냥 다른 사람의 입에 나에 대한 말이 오르내리는 게 싫으니까 (그냥 넘기는 거죠)‧‧‧ 여러 사람의 입에서 오르내리면 왜곡이 되는 건 당연한 거고 이게 더 이상하게 부풀려지고 그러잖아요. (참여자 1)*

*(선배 간호사에게 언어폭력을 당하여) 사직하겠다고 말하자 마자 할 수 있는 것도 아니고 같이 계속 일을 해야 하는 상황이고‧‧‧ 언젠가 볼 수 있는 선생님들인데‧‧‧ 그걸 감당할 자신이 없어서 무서우니까 (아무 말 못하는 거죠)‧‧‧ (참여자 3)*

*‧‧‧(제가 언어폭력에 대해 문제 제기를 하면 분위기가 나빠져서) 이걸로 피해를 제 동기들이나 아래 연차 친구들이 볼까 봐 그 부분이 걱정되어요. 저는 그만두면 그만이지만‧‧‧ 그만둘 각오로 문제화시킬까 (하고) 생각을 했다가도 못할 것 같아요. (참여자 4)*

#### 주제 6: 체념하고 침묵함

참여자들은 환자나 보호자들로부터 당하는 언어폭력은 해결책이 없다고 체념하여 침묵하는 분위기가 간호사들과 조직 전체에 만연해 있다고 하였다. 간호사들이 당한 언어폭력 사건이 만족스럽게 해결이 된 선례가 없고, 환자나 보호자들로부터 언어폭력을 당한 후에도 가해자인 환자를 계속 돌봐야 하는 상황이라 체념할 수밖에 없다고 하였다.

*제가 무언가를 더 한다고 해서. (조직에서) 더 액티브하게 해결해줄 거라는 기대도 없고. 그냥 여기 원래 이런 곳이니까 내가 참아야지‧‧‧ (참여자 4).*

*‧‧‧어차피 해결할 수 없으니까 그냥 나만 입 닫으면 되지, 어차피 내가 이야기를 꺼내 봤자 나만 손해지‧‧‧ 내 입만 아프지‧‧‧ 약간 이런 식으로‧‧‧ 얘기를 해도‧‧‧ (상황이) 개선되는 것이 아니니까‧‧‧ 무슨 일 있었다는 말을 잘 안 하게 되더라구요. (참여자 6)*

*(언어폭력을 당하고도) 울고 나서 일은 해야 하니까‧‧‧ 엄청 바쁜 날이었거든요‧‧‧ 제가 감정을 수습할 틈 없이 일을 해야 할 게 엄청 쌓여 있었으니까‧‧‧ (부서장이) 유급휴가를 줄 수 있다 하긴 했는데‧‧‧ (대체할) 인원도 안 주거든요. 간호사가 그만뒀는데 줄 사람 없다고. (휴가를) 달라고 하기도 민망한 그런 상황이고‧‧‧ (참여자 5)*

### 주제모음 4. 개인부담(個人負擔)

마지막 주제모음에는 ‘큰 상처와 작은 깨달음’과 ‘나를 희생해서 해결하려고 애씀’이 포함되었다. 이는 조직의 무관심과 침묵 속에서 언어폭력 사건이 피해자들의 삶과 직업 경력에 남긴 부정적 결과를 피해 당사자인 간호사 개인이 감당하고 있으며, 언어폭력 사건 발생 시 대처와 예방을 위한 부담 또한 간호사들이 개별적으로 떠안고 있음을 의미한다.

#### 주제 7: 큰 상처와 작은 깨달음

참여자들은 언어폭력 경험이 신체적 건강문제의 발생이나 악화, 또는 미리 걱정하고 극도로 조심하게 되는 성격 변화, 심리적 트라우마, 모든 인간관계가 어려워지거나 누적된 상처로 인한 사직, 사직의도를 가지게 되는 등의 전방위적인 후유증을 남긴다고 하였다. 또한 그런 큰 상처를 경험하는 과정에 다른 사람들의 위로를 받기도 하면서 인간관계의 중요성을 깨닫게 되었다고 하였다.

*(언어폭력을 심하게 당한 후) 잠을 못 자긴 했어요. 새벽에도 병원 꿈 꾸면 거실로 뛰쳐나와서 돌아다닌 적도 있고‧‧‧ (병동에서 함께) 일하는 사람들하고 사이에서는 방어적이라고 해야 되나요? 무슨 말을 하면 꼬투리 잡히고 무슨 말 하면 그걸 또 뒤에서 얘기하고 흉보고 하니까 내 의견을 말을 못하겠고, 음‧‧‧ 편하게 동료라는 느낌을 못 가지는 (거예요)‧‧‧ 사람들하고 깊은 관계를 형성하지는 못하는 것 같아요. (참여자 3)*

*‧‧‧저도 다른 곳에서 서비스를 받을 때 제가 손해 보면 안 된다는 생각이 드는 거예요‧‧‧ 제가 원래는 컴플레인을 하는 성격이 아니었는데‧‧‧ 성향이 바뀌는 것 같아요‧‧‧ (참여자 2)*

*(언어폭력에 대해 말해도) 부모님들은 이해를 못 하시는 거예요‧‧‧ 너만 유독 그러냐‧‧‧ 힘든 것이 있으면 얘기 안 하게 되더라고요‧‧‧ 동기들, 제 편인 동료들이 있어서 일하면서 같이 의지를 하고‧‧‧ (참여자 4)*

*퇴근 후‧‧‧ (병동 동료들을) 따로 안 만나려고 해요‧‧‧ 병원에서의 일은 병원 벗어나면 더 이상 상관하기가 싫어서‧‧‧ (선배나 동료 간호사들과) 마음이 잘 맞아서 지내다가도 제가 어느 순간 말 실수할 수 있는 상황도 만들어지는 여지가 많아져서‧‧‧ 평소에 알고 있던 친구들이랑만 생활하려고 해요. (참여자 5)*

#### 주제 8: 나를 희생해서 해결하려고 애씀

이 주제는 언어폭력 사건을 경험한 후 대처와 예방을 위한 노력을 의미한다. 참여자들은 할 말을 다 하지 못해 억울해하고 속앓이를 하면서도 가해자에 대해 억지로 이해하려고 애쓰며, 마음에도 없는 사과로 상황을 모면하거나 상대의 부당한 요구를 들어주고 넘어갈 수밖에 없다고 하였다. 또한 ‘잊으려고 애쓰거나 생각을 바꿔 좋은 면을 보려고 노력하면서 자기개발을 하는 등’ 스스로를 바꾸려는 노력을 통해 문제에 대처하고 있다고 하였다.

*(의사, 동료 간호사들은) 일할 때 다들 바쁘니까, (환자들은) 아프니까‧‧‧, 하고 상대방을 배려하고 이해하려는 게 습관이 되어 버린 것 같네요. 처음에는 열 받지만 다 받아들이고 일하려고‧‧‧ (참여자 2)*

*제가 머리를 숙이지 않으면 해결되지 않더라고요‧‧‧ 제가 잘못하지 않았어도 ‘죄송해요.’라고‧‧‧ (간호사들 사이에) ‘니가 죄송하다고 하면 좀 괜찮아’ 약간 이런 생각들이 있더라고요. (참여자 6)*

*여긴 원래 이런 곳이니 내가 참아야지, 내가 이해해야지‧‧‧ 뭔가 너무 제 주장을 하고 제 이야기를 너무 하면 그게 오히려 독이 되는 것 같아요. (마음 속으로) 참자 그냥 내가 넘어가자‧‧‧ (참여자 4)*

*(제가) 흥분하거나 성격이 급한 스타일인데, 그걸 조금씩 고쳐 나가고 있는 것 같아요. 제가 좀 바빠지거나 부당한 대우를 받거나 하면 조금 예민해지는 경우가 있더라고요‧‧‧ 일하다 보니 알게 된 제 성격인데 고쳐 나가려고 하고‧‧‧ 의사들이 무시하는 듯한 발언을 할 경우에는 내가 무시 받지 않으려면 더 많이 알고 더 공부해야 하고 더 일을 잘 해야겠다(하고 생각하고)‧‧‧ (참여자 2)*

## Discussion

본 연구는 의료기관에 근무하면서 언어폭력 피해를 입은 임상 간호사들의 경험을 현상학적 연구방법을 적용하여 총체적이고 심층적으로 이해하고자 하였다. 분석된 주제모음과 주제를 중심으로 연구 결과를 논의하고자 한다.

첫째, ‘빙산일각(氷山一角)’은 임상 간호사들이 직장 내에서 일상적으로 언어폭력에 노출되고 있지만 공론화되는 사건은 극히 일부에 불과함을 의미한다. 이는 국내•외 선행연구에서 일관되게 나타나고 있는 현상이다[1,16]. 특히 본 연구의 참여자들은 간호사에 대한 언어폭력의 가해자는 환자, 보호자, 동료 간호사, 의사, 불특정 가해자 등 다양한 데 비해 사회적 관심은 간호사들 사이의 태움에만 초점을 두고 있고 있다고 지적하였다. 환자, 보호자로부터 가해지는 언어폭력에 대한 관심과 연구가 필요함을 시사하는 대목이다.

주목할 점은 본 연구 참여자들이 말하는 언어폭력 상황에는 부적절한 호칭과 반말, 고함, 욕설 등 잘못된 언어의 사용뿐 아니라 비언어적 메시지도 포함되었고, 밀치거나 머리채를 잡는 등의 물리적 폭력도 자주 동반된다는 점이다. 이는 언어폭력의 위험성을 보여주는 한편으로 개념상 언어적 학대의 범주나 선행연구[5]에서 정의하는 언어폭력과도 차이가 있다. 추후 연구를 통해 임상 간호사들의 경험을 반영하여 직장 내 폭력과 언어폭력의 개념을 명확히 정의할 필요가 있다고 생각된다. 아울러 언어폭력이 다른 제반 폭력을 동반할 수 있음에 경각심을 갖고 보다 적극적이고 신속한 대응방안을 마련하는 것이 중요하다.

둘째, ‘상황귀인(狀況歸因)’에서는 언어폭력이 개별 간호사의 행위적 문제가 아니라 예민하고 심리적으로 취약한 상태에 놓인 환자와 보호자를 돌보면서 다양한 사람들과 협력해야 하는 직무 특성, 간호사에 대한 잘못된 인식과 열악한 근무환경과 관련되어 나타나기 때문에 해결방안도 조직적, 사회적 차원에서 모색되어야 한다는 공통된 인식이 확인되었다. 이러한 결과는 언어폭력이 간호사를 전문가로 보지 않고 무시하는 사회적 분위기, 가해자 자신의 문제를 간호사에게 투사하여 화풀이 대상으로 생각하는 점[23], 직원이나 환자의 행동, 병원 환경, 전문직 역할과 책임 및 대기 시간 등의 여러 요인이 상호 작용한 결과[24]라는 선행연구 결과들과 일치한다. 이는 물리적 환경 개선 등 전반적인 질 향상 정책을 통해 직장 내 폭력을 감소시키고자 만들어진 OSHA의 가이드라인[25]이 국내에서도 유효할 수 있음을 시사한다. 이러한 제도적 접근과 함께 간호사의 직무 특성을 반영한 언어폭력 예방 및 대처 전략 가이드라인 개발, 간호사에 대한 사회적 인식을 개선하기 위한 노력이 동반되어야 할 것이다.

셋째, ‘두려움과 체념’에서는 Van Dyne 등[26]이 분류한 체념적 침묵(acquiescent silence), 방어적 침묵(defensive silence), 친사회적 침묵(prosocial silence) 중 체념적, 방어적 침묵이 특징적으로 나타난 점에 주목할 필요가 있다. 체념적 침묵은 발언해도 소용없다는 비관적 동기, 방어적 침묵은 발언으로 인해 부정적인 결과를 초래할까 봐 자기보호적 동기에서 선택되는 침묵이다[27]. 이로 인해 간호사들이 폭력을 업무의 일부로 받아들이고, 폭력 사건을 예방할 책임이 돌봄 제공자에게 있다고 생각하는 사람들이 많아 비난받을 것을 우려하여 잘 보고하지 않게 되며[27,28], 결과적으로 조직에 부정적 영향을 초래하게 된다[29].

이러한 조직침묵은 상사의 태도에 따라 달라지며, 수직적, 수평적 의사소통이 원활할수록 감소한다[30,31]. 또한 가해자에 대한 처분 없이 폭력은 근절될 수 없으며[28], 의료종사자에 대한 폭력을 해결하기 위한 가장 중요한 해결책은 폭력 사건에 대한 보고와 안전한 환경 조성에 있다고 하였다[32]. 이에 미국간호사협회는 판단력이 떨어지거나 정신 상태가 좋지 않은 환자를 대할 때에도 간호사를 안전하게 보호하는 것이 중요하다고 강조하면서 직장폭력에 대한 무관용 정책을 지지하는 시민 캠페인을 진행하고 있다[33]. 국내에서도 언어폭력에 대해 드러내어 이야기할 수 있는 분위기를 조성하고, 병원에서 발생하는 모든 유형의 언어폭력 사건을 보고하도록 교육하여 확실한 문제 해결을 하는 시스템을 구축할 필요가 있다.

넷째, ‘개인부담(個人負擔)’은 언어폭력을 경험한 간호사들에 대한 조직 차원의 대응과 지원이 부족하여 언어폭력에 대한 대처나 언어폭력으로 인한 피해도 개인적으로 감당하고 있음을 의미한다. 임상 간호사들이 언어폭력 경험 시 참고 견디는 것, 주변 사람에게 부당함을 하소연하는 것 외에도 도움을 요청하고 맞서 대응하는 등의 전략을 채택하고 있는 것으로 나타난 선행 연구[16,34]에 비하여 보아도 본 연구의 참여자들은 훨씬 소극적인 태도를 보였다. 본 연구 참여자들의 근무경력이 평균 37.7개월로 짧은 것과 관련이 있는 것으로 생각되는데, 이들은 조직에 대한 기대가 낮으면서도 조직 내 인간관계를 중시하고 조직 분위기나 풍토를 중요하게 생각하고 있는 것을 확인할 수 있었다.

한편, 의료기관 내 언어폭력에 대한 대처를 위한 국내 연구는 자기주장 강화 훈련[35], 회복력 증진을 위한 폭력대처 프로그램 등[36] 개인의 대처 능력을 증진하는 프로그램에 초점을 두었는데, 정부가 연구를 주도하여 정책에 반영하고 가이드라인을 제시하는 외국 사례를 참고할 필요가 있다[24]. 미국의 경우 국립직업안전위생연구소(National Institute for Occupational Safety & Health)에서 전 지역 직장 내 폭력에 대한 사건, 사고 통계자료를 분석, 관리할 뿐만 아니라 간호사를 대상으로 직장 내 폭력 예방을 위한 교육 훈련 프로그램을 운영하고 있다[37].

국내에서도 개인들의 대처 능력 향상에서 더 나아가 언어폭력을 포함한 직장 내 폭력 사건 보고의 활성화, 통계 관리 및 분석을 통한 재발 방지책 마련, 실효성 있는 폭력 대응 매뉴얼 구축을 위한 조직적, 사회적 차원의 노력이 필요하다. 아울러 간호사에 대한 인식 및 태도 개선 활동, 산업재해 통계 관리 시 재해유형에 언어폭력을 포함하여 현황을 파악하고 관리하는 등 사회적 노력이 필요하다. 이를 위하여 언어폭력의 개념을 분명히 정의하여 폭력의 형태나 사건의 경중을 가리지 않고 모두 보고될 수 있는 체계를 구축하는 것이 우선되어야 할 것이다.

이상과 같이 본 연구에서 임상 간호사들이 경험하는 언어폭력은 잘 드러나지 않는 경향이 있어 드러난 사실은 극히 일부분에 지나지 않고, 발생하는 상황도 간호사 개인의 대처보다는 상황적 요인에서 비롯됨을 확인할 수 있었다. 그럼에도 불구하고 조직 전반에 간호사들의 언어폭력에 대한 침묵하는 태도가 지배적이어서 간호사들이 개인적으로 대응하고 있는 것으로 확인되었다. 간호사들에 대한 언어폭력을 줄이기 위해서는 간호사들이 보다 적극적으로 이를 드러내고 사회적 차원의 인식 개선 활동을 전개할 필요가 있음을 보여준다.

이 연구에는 5년차 미만의 경력이 낮은 간호사들이 참여하였고, 참여자들의 부서가 일반병동, 외래, 중환자실로 국한되어 임상 간호사 전체의 경험을 설명하기에는 제한이 있다. 그러나 간호사들이 인식하는 언어폭력의 심각성, 언어폭력이 사회적, 구조적 요인에서 기인한다는 점을 확인하였다는 점에 의의가 있다.

이러한 결과를 바탕으로 의료기관 내에 간호사들이 경험하는 언어폭력을 포함하여 직장폭력 보고 체계를 만들고, 신입간호사 교육, 간호사리더십 교육 프로그램 등에 언어폭력에 대한 대처 방안을 포함하는 근거로 활용할 수 있을 것이다.

## Figures and Tables

**Table 1.** Participants’ characteristics

No.	Age (year)	Employment period (month)	Workplace	Hospital level	Turnover experience	Living with
1	27	51	General ward	General	Yes	Alone
2	26	40	General ward	Tertiary	No	Alone
3	27	48	General ward+	General	Yes	Spouse
			outpatient clinic			
4	24	18	General ward	Tertiary	No	Parents, siblings
5	25	28	General ward	General	Yes	Alone
6	27	41	General ward+	Tertiary	Yes	Parents
			intensive care unit			

**Table 2.** Codes, themes, and theme clusters

Code	Theme	Theme cluster
• Feeling of being ignored and insulted	Various types of verbal violence	Tip of the iceberg
• Feeling of becoming the unjust target of anger		
• Feeling of being unfairly rebuked		
• Feeling of intended harassment		
• Feeling threatened		
• Exposed everyday	Too common, taken for granted	
• Not considered a big deal		
• Unspecified number of perpetrators		
• Behaviors not recognized as violence		
• Not seen as an expert	Not because of my fault.	Beyond me and my control
• Feeling sandwiched between job characteristics		
• Poor working conditions		
• Problems beyond one’s personal control	Out of my control	
• Unable to cope alone		
• Fear of what will happen if disclosed	Silence due to fear	Fear and resignation
• Fear of being considered annoying		
• Fear I will be unsupported		
• Decisions beyond my will	Silence due to resignation	
• Very scarce support for victims		
• Never had a satisfactory resolution		
• Persistent misperceptions about nurses		
• The remaining aftermath of huge damage	Small realization after big damage	Personal burden
• Realizing the importance of relationships		
• Feeling not fully understood by anyone	Trying to solve it by sacrificing myself	
• Just trying to understand		
• Trying to change myself more than anything else		
• Accepting unreasonable requests		
